# The global prevalence of emotional eating in overweight and obese populations: A systematic review and meta‐analysis

**DOI:** 10.1111/bjop.12768

**Published:** 2025-01-15

**Authors:** Han Shi Jocelyn Chew, Rou Yi Soong, Wei How Darryl Ang, Jia Wen Ngooi, Jiyoung Park, Jenna Qing Yun Ow Yong, Yong Shian Shawn Goh

**Affiliations:** ^1^ Alice Lee Centre for Nursing Studies, Yong Loo Lin School of Medicine National University of Singapore Singapore Singapore; ^2^ Yong Loo Lin School of Medicine National University of Singapore Singapore Singapore; ^3^ Global Nursing Research Centre, Graduate School of Medicine University of Tokyo Tokyo Japan; ^4^ School of Nursing Inje University Gimhae South Korea; ^5^ Health and Social Sciences Cluster Singapore Institute of Technology Singapore Singapore

**Keywords:** emotional eating, health, obesity, prevalence, weight

## Abstract

This systematic review aims to investigate the current prevalence of emotional eating and its associated factors in overweight and obese populations. We included studies that (1) reported prevalence of emotional eating; (2) were in the context of weight gain or overweight and obesity; (3) used a validated psychometric tool to assess emotional eating; (4) were published as an internationally referred journal article and (5) were reported in the English language. Articles were searched on eight electronic databases (CINAHL, EMBASE, PsychINFO, ProQuest, PubMed, Scopus, The Cochrane Library and Web of Science) from the journals' inception to 11 April 2024. A total of 18 studies, representing a total of 21,237 people, were included in the review. Our study suggested that emotional eating is significantly prevalent at 44.9%. High heterogeneity observed (*I* ^2^: 98.7%) can be attributed to differences in measurement tools for emotional eating, but not differences in geographical regions. By providing insight to the current prevalence of emotional eating and its relevant factors, this study outlines the steps to take in future research and practice to tackle emotional eating and related health issues like obesity. There is a need to develop standardized measurement tools for emotional eating, and further investigate sociodemographic factors.

## BACKGROUND

Emotional eating, characterized by the consumption of food in response to emotional cues rather than physiological hunger, has emerged as a pervasive behavioural phenomenon associated with obesity and obesity‐related cardiometabolic disorders such as diabetes, hypertension and hyperlipidaemia (Frayn & Knäuper, 2018; Gibson, 2012; van Strien et al., 2007). Emotional eating has also been associated with mental health conditions such as depression, anxiety, and disordered eating patterns, highlighting its role in the complex interplay between psychological well‐being and physical health (Dakanalis et al., 2023).

The complexity of *addressing* emotional eating as a maladaptive eating behaviour lies in the bidirectional relationship between emotional triggers and weight changes, creating a vicious cycle (Djalalinia et al., 2015). For instance, negative emotions may trigger episodes of overeating, which creates feelings of guilt or shame that trigger comfort eating, leading to weight gain that again triggers a vicious cycle of emotional eating (Burnatowska et al., 2023).

Moreover, emotional eating is influenced by complex biopsychosocial factors, including individual psychological profiles, sociocultural contexts, and environmental triggers.

Various theories have been proposed to explain emotional eating, including the psychosomatic theory, affect regulation model, escape theory, and restraint theory (Spoor et al., 2007). For instance, negative emotions such as stress have been reported to trigger emotional eating by increasing ghrelin levels, a stomach peptide that signals hunger and modulates the brain's reward centre, promoting the selection of rewarding comfort foods that are usually calorie‐dense unhealthy foods (Abizaid, 2009; Meye & Adan, 2014).

Despite its widespread occurrence and impact, comprehensive data on the global prevalence of emotional eating remains sparse. Existing studies often focus on specific populations, such as adolescents, women, or individuals with obesity, limiting the generalizability of findings. Additionally, the methods used to assess emotional eating, including self‐report questionnaires and behavioural experiments, vary widely, contributing to inconsistencies in prevalence estimates. These gaps underscore the need for standardized approaches to studying emotional eating and for research that spans diverse geographical and cultural contexts.

This manuscript aims to synthesize current evidence on the global prevalence of emotional eating, with a focus on regional variations, demographic differences, and methodological challenges. By providing a comprehensive overview of the existing literature, this study seeks to bridge knowledge gaps and inform future research and intervention efforts.

## METHODS

This systematic review was registered with PROSPERO (CRD42023433583) and reported according to the Preferred Reporting Items for Systematic reviews and Meta‐Analyses (Page et al., 2021) checklist (Table S1; Page et al., 2021). Two reviewers (HSJC, RYS) independently performed the study selection, data extraction and methodological quality appraisal. Discrepancies were resolved through discussions with a third reviewer (GYSS). The Cohen's kappa statistic (κ) was used to assess the inter‐rater reliability (perfect agreement = 1.00–0.81; substantial = 0.80–0.61; moderate = 0.60–0.41; fair = 0.40–0.21; none to slight agreement = 0.20–0.01; McHugh, 2012).

### Eligibility criteria and study selection

**FIGURE 1:**
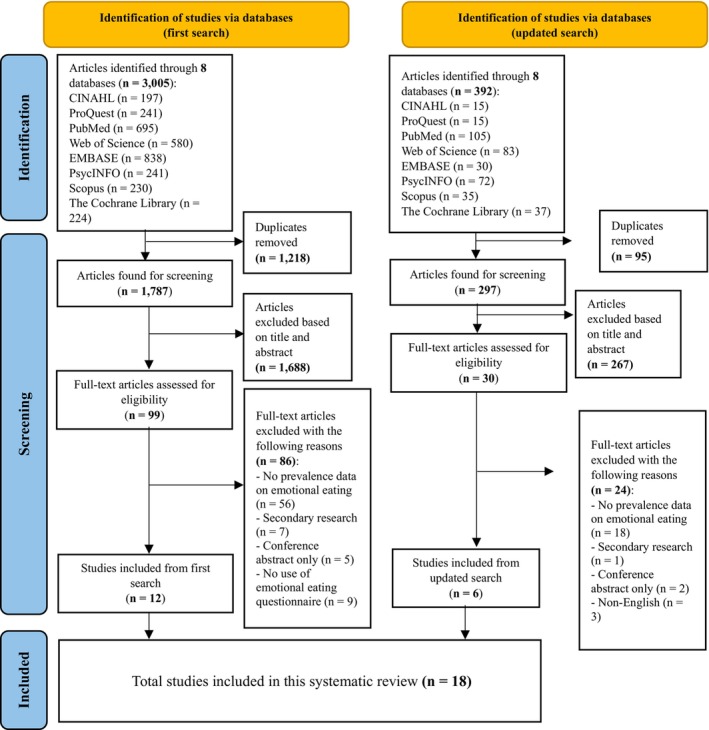
PRISMA 2020 flow diagram of review selection for systematic review.

Studies were included if they (1) reported the prevalence of emotional eating; (2) were in the context of weight gain or overweight and obesity; (3) used a validated psychometric tool to assess emotional eating; (4) were published as an internationally referred journal article and (5) were reported in the English language. Studies were excluded if they used a validated psychometric tool to assess emotional disturbances (e.g. depression) and not eating behaviours. Citations were managed using the EndNote X20 software and the study selection process is depicted in Figure 1.

### Search strategy

A search for existing similar reviews was first conducted in the Cochrane Library, Google Scholar and PROSPERO to prevent duplication. The search strategy was developed iteratively based on index terms (e.g. Medical Subject Heading terms) and keywords. Keywords from similar studies were retrieved to build the search strategy. Keywords included emotional, stress, boredom, overweight, obesity, obese, weight gain, weight loss, weight maintenance, body mass index, prevalence, incidence, proportion and epidemiology. The search strategy for each database is detailed in Table S2.

A three‐step search was conducted to locate eligible articles (Cumpston et al., 2022). First, a search was conducted in eight electronic databases (i.e. CINAHL, EMBASE, PsycINFO, ProQuest, PubMed, Scopus, The Cochrane Library and Web of Science) from the journals' inception to 10 January 2023. An updated search was conducted on 11 April 2024. Next, after trials were retrieved from clinical registries, the authors of eligible trials were contacted to see if their trials were published. Third, a hand search of the reference list of included studies was performed to maximize the search. An email was sent to the authors to obtain further information when there was insufficient information in the trial registry or publication.

### Data extraction

A data collection form was created using Microsoft Excel and pilot‐tested on five articles where no changes were needed. The following information was extracted: author, publication year, country, sample size, study design, sample characteristics, mean age, the proportion of males, education level with the highest proportion and corresponding proportion, income level with the highest proportion and corresponding proportion, emotional eating instrument, cut‐off score for overweight and obesity, the proportion of people with overweight and obesity, the proportion of people with emotional eating and proportion of people with emotional disturbances (e.g. anxiety and depression).

### Methodological quality appraisal

The included articles were assessed for methodological quality using the Joanna Briggs Institute's (JBI) Critical Appraisal Checklist for Studies Reporting Prevalence Data, which contains nine items rated as Yes, No, Unclear or Not Applicable (Munn et al., 2015). Score were computed as a percentage of the total score, where items identified as Not Applicable were excluded.

### Data synthesis

Statistical analyses were performed using R (version 4.1.3; Team, 2022). The prevalence and corresponding standard errors (SE) of emotional eating were calculated using the equations below:

$$\text{prevalence}\;\left(p\right)=\frac{\text{number of people with emotional eating}\;\left(k\right)}{\text{total sample size}\;\left(n\right)}$$

$$\mathrm{SE}=\sqrt{\frac{p\left(1-p\right)}{n}}$$

As the precision of proportion estimates tended to be overestimated due to the compressed standard error values of proportions near to 0 and 1, proportion estimates were transformed using the logit link before pooling (Lin & Xu, 2020). Given the potential heterogeneity and small sample size of prevalence data, meta‐analyses were conducted using random effects models with generalized linear mixed‐effects model and Hartung‐Knapp‐Sidik‐Jonkman adjustment to reduce the chance of false positives (Van Aert & Jackson, 2019). Between‐study heterogeneity was examined using *Q* statistics and interpreted in *I* ^2^ statistics where ≥75% (considerable), 50%–90% (substantial), 30%–60% (moderate), and 0%–40% (unimportant; Higgins & Li, 2022; Lin, 2020). Sensitivity analysis was performed using the leave‐one‐out method and publication bias was assessed using Egger's regression tests and funnel plots symmetry (Sterne et al., 2011). Subgroup analyses were conducted on a priori factors including measurement tool, geographical region, mean age, proportion of males, proportion of overweight and obesity, and risk of bias rating.

## RESULTS

Our initial search (January 2023) yielded 3005 citations, of which 1218 duplicated citations were removed. 1787 titles and abstracts were screened, of which 99 full‐text articles were assessed for inclusion eligibility. A total of 86 articles were excluded. The updated search yielded 392 citations, of which 95 duplicates were removed. The remaining 297 had their titles and abstracts screened. From there, 30 full‐text articles had to be assessed for inclusion eligibility and 24 articles were excluded in total and the reasons for exclusion are detailed in Table S3.

A total of 18 articles were included in this review, representing 21,237 people with a mean age of 32.4 years old and an of average proportion of 34.1% males (*n* = 18) (Table 1). The average proportion of people with overweight and obesity was 41.5% (*n* = 13). Only four studies reported the corresponding prevalence of anxiety and depression (Al‐Musharaf, 2020; Chacko et al., 2015; Constant et al., 2018; Sze et al., 2021). One study reported the prevalence of anxiety, but not depression (Bizjak & Adamič, 2023).

**TABLE 1:** Characteristics of the 18 studies included in this review.

Authors, year	Country	Study design	*N*	Sample characteristics	Mean age, % male	Education level with highest proportion (%)	Income level with highest proportion (%)	% with emotional eating, measurement tool	% with overweight and obesity (cut‐off score)	% with anxiety, depression
Al‐Musharaf (2020)	Saudi Arabia	Cross‐sectional	638	University students	22, 0.0	Bachelor (64.7)	Household monthly income >20,000 Saudi Riyals (39.8)	47.5, EES	Obese: 9.1 Overweight: 18.1 (Obesity: ≥30 kg/m^2^ Overweight: 25–29.9 kg/m^2^)	27.0, 42.8
Chacko et al. (2015)	USA	Cross‐sectional	337	Primary care patients with obesity	48.7, 31.0	≤High school (36.0)	$20,001–$40,000 (36.0)	48.7, TFEQ‐R18	100.0 (BMI ≥ 35 kg/m^2^)	44.0 (depression or anxiety)
Constant et al. (2018)	France	Cross‐sectional	335	Female university students	20.1, 0.0	NR (NR)	NR (NR)	29.4, TFEQ‐ R18	0.0 (BMI ≥ 25)	51.3, 44.8
Barak et al. (2021)	USA	Cross‐sectional	5863	General population	50.8, 29.7	College (45.6)	Annual household salary of >$70,000 (37.3)	20.5, Adapted DEBQ	33.6 (BMI ≥ 30)	NR, NR
Grajek et al. (2022)	Poland	Cross‐sectional	300	University students	26, 38.8	University (100.0)	Non‐permanent income (86.9)	37.9, TFEQ‐13	Obese: 7.4 Overweight: 64.7 (Obesity: ≥30 kg/m^2^ Overweight: 25–29.9 kg/m^2^)	NR, NR
Jääskeläinen et al. (2014)	Finland	Prospective cohort study	6945	16‐year‐olds	16, 51.8	High school (100.0)	NR (NR)	29.7, Ways of Coping Checklist	Obese: 3.6 Overweight: 11.8 (Obesity: ≥28.88 kg/m^2^ Overweight: 23.90–28.87 kg/m^2^)	NR, NR
Mohapatra et al. (2021)	India	Cross‐sectional	607	General population	36.3, 48.4	Postgraduate (53.2)	NR (NR)	42.2, EEQ	NR (NR)	NR, NR
Schnettler et al. (2021)	Chile	Cross‐sectional	884	Women	40.8, 0.0	Technical (36.8)	Low (34.3)	21.9, DEBQ	Obese: 28.8 Overweight: 40.0 (NS)	NR, NR
Skolmowska et al. (2022)	Poland	Cross‐sectional	1126	Adolescents	16.7, 27.4	High school (100.0)	NR (NR)	38.8, EEQ	Obese: 7.7 Overweight: 14.0 (WHO growth reference – Overweight: BMI ≤ 85th–95th percentile; and Obesity: BMI ≥ 95th percentile)	NR, NR
Sze et al. (2021)	Hong Kong	Cross‐sectional	424	University students	20.2, 47.4	University (100.0)	No income (48.1)	9.9, DEBQ	26.6 (BMI > 23 kg/m^2^)	9.9 (moderate) 1.4 (severe) 3.8 (extremely severe), 9.9 (moderate) 3.3 (severe) 0.5 (extremely severe)
Tuncer and Çetinkaya (2020)	Turkey	Cross‐sectional	130	Patients with severe mental disorder	41.3, 69.6	NR (NR)	NR (NR)	49.2, TFEQ‐R21	79.2 (NS)	NR, NR
Wong et al. (2020)	Australia	Cross‐sectional	387	Patients with obesity	51.7, 29.0	NR (NR)	NR (NR)	58.0, EES	0.4 (BMI ≥ 40)	NR, NR
Aagaard et al. (2023)	Denmark	Cross‐sectional	184	Children with overweight/obesity	11.8, 39.1	NR (NR)	200,000–499,999 DKK (45.7)	74.1, CEBQ	94.6 (BMI‐SDS > 1 SD)	NR, NR
Bizjak and Adamič (2023)	Slovenia	Cross‐sectional	1487	General population	33.9, 9.1	NR (NR)	NR (NR)	37.7, DEBQ	Obese: 16.6 Overweight: 25.1 (Obesity: ≥30 kg/m^2^ Overweight: 25–29.9 kg/m^2^)	31, NR
Gaździńska et al. (2024)	Poland	Cross‐sectional	760	Polish Air Force	39.4, 92.1	>Secondary education (83.6)	NR (NR)	12.3, EEQ	Obese: 15.0 Overweight: 48.4 (Obesity: ≥30 kg/m^2^ Overweight: 25–29.9 kg/m^2^)	NR, NR
Constant et al. (2023)	France	Cross‐sectional	302	Female university students	20.9, 0.0	University (100.0)	NR (NR)	91.4, EOQ	Obese: 2.6 Overweight: 10.3 (Obesity: ≥30 kg/m^2^ Overweight: 25–29.9 kg/m^2^)	NR, NR
Hawash et al. (2024)	Egypt	Cross‐sectional	200	Older adults	65.3, 51.0	University (35.5)	Sufficient to some extent (48.5)	98.0, EEQ	Obese: 54.0 Overweight: 27.0 (Obesity: ≥30 kg/m^2^ Overweight: 25–29.9 kg/m^2^)	NR, NR
Zuhair et al. (2024)	Pakistan	Cross‐sectional	328	University students	21, 48.8	University (100.0)	NR (NR)	39.0, TFEQ‐R21	NR (NR)	NR, NR

Abbreviations: BMI, Body Mass Index; BMI‐SDS, Body Mass Index Standard Deviation Score; CEBQ, Child Eating Behaviour Questionnaire; DEBQ, Dutch Eating Behaviour Questionnaire; DKK, Danish Krone; EEQ, Emotional Eater Questionnaire; EES, Emotional Eating Scale; EOQ, Emotional Overeating Questionnaire; NR, Not Reported; NS, Not Specified; SD, Standard Deviation; TFEQ, Three‐Factor Eating Questionnaire; USA, United States of America; WHO, World Health Organization.

### Quality assessment

The findings of the quality assessment can be found in Table S4. The overall scores of the quality assessment ranged from 56% to 100%. All studies were rated ‘yes’ in criterion four and eight. Most of the included studies did not have appropriate or clear sampling strategies (67%). Half of the studies (50%) did not carry out data analysis with sufficient coverage and half (50%) had inadequate response rates that were not addressed. None of the studies were excluded based on the quality assessment results.

### Prevalence of emotional eating

**FIGURE 2:**
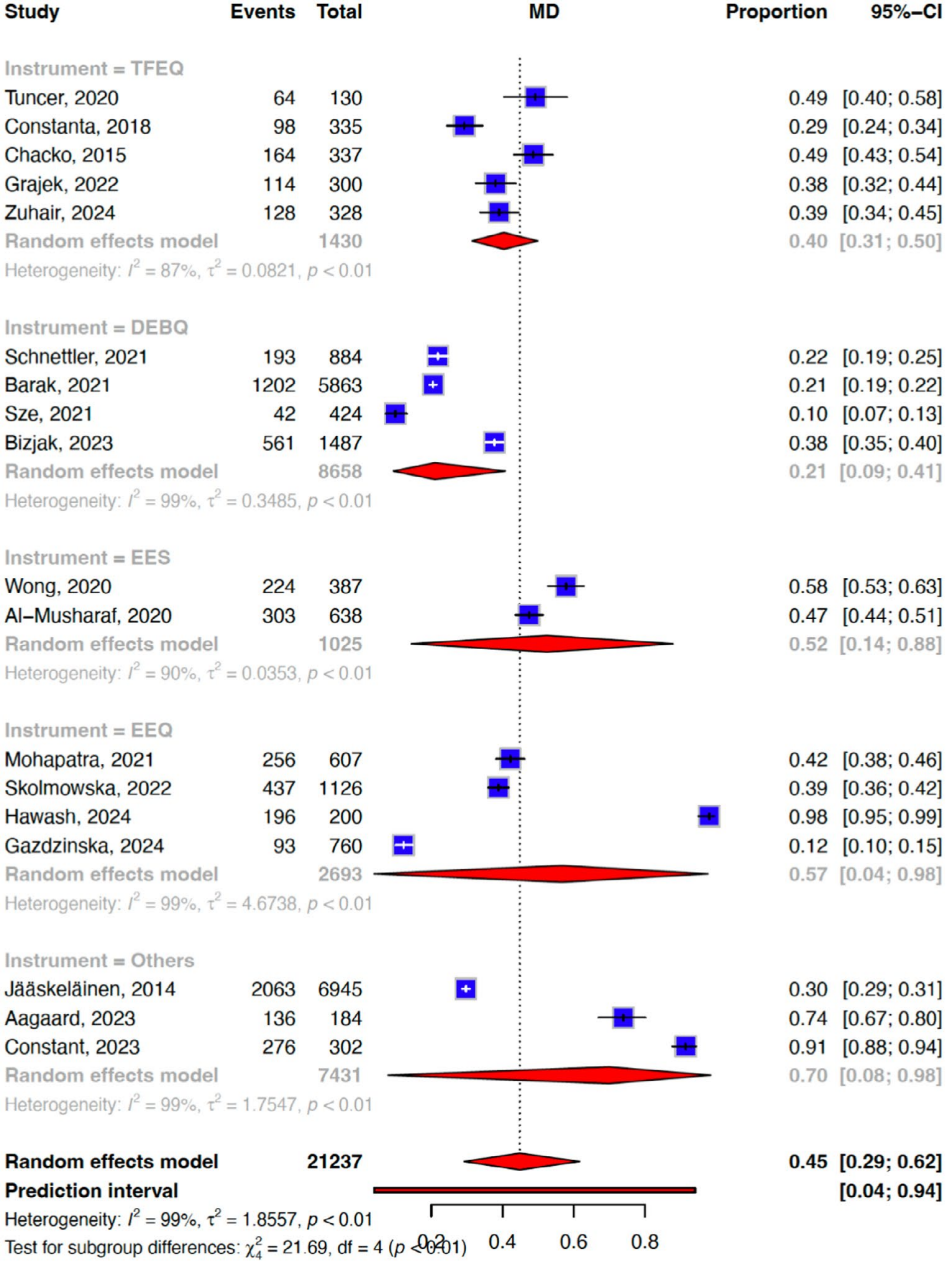
Forest plot depicting the meta‐analysis and subgroup analysis of the pooled prevalence of emotional eating based on the instrument used to measure emotional eating.

**FIGURE 3:**
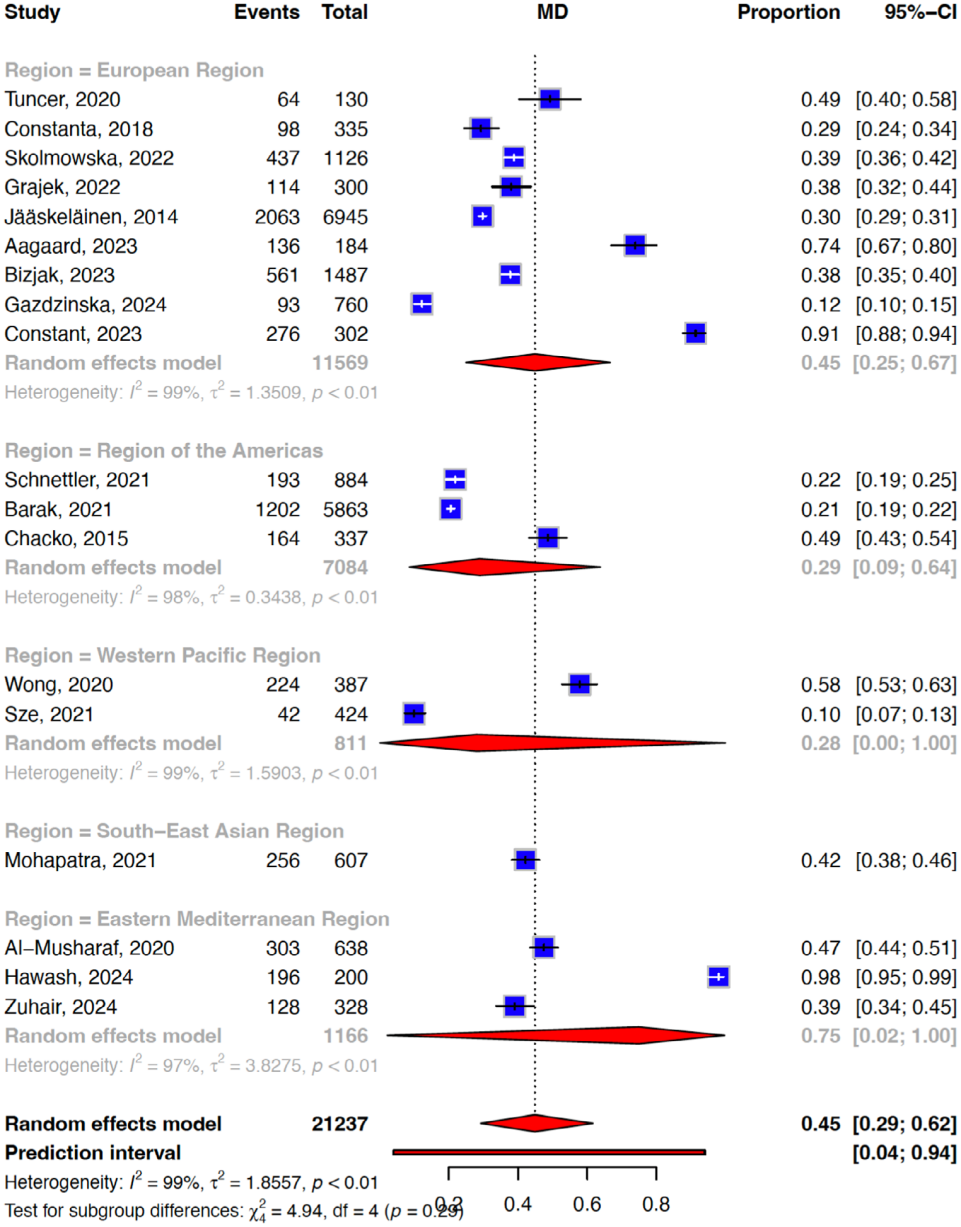
Forest plot depicting the meta‐analysis and subgroup analysis of the pooled prevalence of emotional eating based on the region where the study was conducted.

Our meta‐analysis of 18 unique effect sizes of the included studies suggested that the prevalence of emotional eating was 44.9% (95% confidence interval (CI) = 0.29–0.62, *I* ^2^: 98.7%). The heterogeneity observed could be explained by the emotional eating measurement tool used (*Q* = 403.7, df: 6, *p* < .001) (Figure 2) but not by the geographical region where the study was conducted (*Q* = 4.94, df:4, *p* = .293; Table 2, Figure 3). No significant moderation effect was found for mean age (*t* = 1.07, df = 16, *p* = .30) proportion of males (*t* = −0.64, df = 16, *p* = .53), proportion of overweight and obesity (*t* = 0.01, df = 10, *p* = .94) (*n* = 12) and risk of bias (*t* = −0.62, df = 16, *p* = .61). Funnel plot asymmetry (Figure S5) and eggers' test (7.62, 95% CI: −13.7‐2.44, *t* = 2.43, *p* = .03) indicated a risk of publication bias.

**TABLE 2:** Subgroup analysis results of the 18 included studies.

Outcomes and subgroups	*n*	MD (95% CI)	*τ* ^*2*^	*I* ^2^, %	Subgroup differences *Q*, *p*‐value
Emotional eating measurement tools					21.7, <.001
TFEQ	5	0.40 (0.31, 0.50)	0.08	87.3	
DEBQ	4	0.21 (0.09, 0.41)	0.35	98.7	
EES	2	0.52 (0.14, 0.88)	0.04	90.3	
EEQ	4	0.57 (0.04, 0.98)	4.67	98.8	
Others (Ways of coping checklist, CEBQ, EOQ)	3	0.70 (0.08, 0.98)	1.75	99.4	–
Region					4.94, .29
European Region	9	0.45 (0.25, 0.66)	1.16	98.5	
Region of the Americas	3	0.29 (0.09, 0.64)	0.59	98.5	
Western Pacific Region	2	0.28 (0.00, 1.00)	1.26	99.4	
South‐East Asian Region	1	0.42 (0.38, 0.46)	–	–	–
Eastern Mediterranean Region	3	0.75 (0.02, 1.00)	1.26	97.2	–

Abbreviations: CEBQ, Child Eating Behaviour Questionnaire; CI, confidence interval; DEBQ, Dutch Eating Behaviour Questionnaire; EEQ, Emotional Eater Questionnaire; EES, Emotional Eating Scale; EOQ, Emotional Overeating Questionnaire; *I* ^2^, statistical measure of study heterogeneity; MD, mean difference; TFEQ, Three‐Factor Eating Questionnaire; *τ* ^*2*^, between‐study variance.

## DISCUSSION

### Prevalence of emotional eating

Based on our meta‐analysis of 18 studies representing 21,237 individuals, we found a 44.9% (95% CI: 29%–62%) prevalence of emotional eating. However, heterogeneity was found to be high (*I* ^2^: 98.7%), potentially due to the difference in measurement tools used for emotional eating and less so due to differences in weight status or geographical regions. With rising rates of non‐communicable diseases and mental health disorders, addressing emotional eating as a modifiable risk factor of cardiometabolic diseases offers a valuable opportunity to improve health outcomes on a population level. Understanding the global prevalence of emotional eating is essential for designing targeted interventions and public health strategies. By identifying populations at higher risk and elucidating the factors that drive emotional eating in different settings, researchers and policymakers can develop culturally sensitive approaches to mitigate its impact.

### Measurement tools

Similar to previous studies (Brantley et al., 2023), the high heterogeneity in our meta‐analysis could be explained by the differences between commonly used tools like the Three‐Factor Eating Questionnaire (TFEQ), Dutch Eating Behavior Questionnaire (DEBQ) and Emotional Eating Scale (EES). These tools differ in their conceptualization of emotional eating, the range of emotions assessed and their scoring systems, all of which contributed to variability in reported prevalence.

For instance, the TFEQ and DEBQ primarily focus on negative emotions, but the DEBQ provides a broader range of emotional triggers while EES delves into subtypes of negative emotions, offering a granular perspective but excludes positive emotions (Schneider et al., 2012) Additionally, measurement methods may vary in their sensitivity and specificity, introducing potential biases (Luijken et al., 2019). For instance, tools with broad definitions may capture transient or context‐specific behaviours, inflating prevalence estimates, while stricter definitions may exclude individuals who engage in emotional eating less frequently but still significantly.

These methodological differences directly influence the pooled estimates in a meta‐analysis, increasing heterogeneity and complicating the interpretation of global trends. Addressing this issue requires careful selection of comparable studies, subgroup analyses based on the measurement tools used, and acknowledging these limitations when drawing conclusions from the data.

### Geographical regions

Based on our subgroup analysis, we did not find a moderation effect of geographical regions on the prevalence of emotional eating. This is contrary to prior studies suggesting that geographical differences could influence cultural, socioeconomic, environmental and dietary factors that influence eating behaviours (Liu et al., 2021; Qian et al., 2013, 2022). For instance, in some cultures, food is closely tied to celebrations and social bonding, potentially increasing emotional eating related to positive emotions (Ljubičić et al., 2023). In others, eating may be more utilitarian, with emotional eating primarily tied to negative emotions.

Triggers for emotional eating may differ between low‐ and middle‐income countries (LMICs) and high‐income countries (HICs) due to various differences such as the availability of calorie‐dense comfort food and the prevalence of emotional eating‐associated mental health disorders such as anxiety and depression (López‐Cepero et al., 2020). Differences in the stigma surrounding emotional eating or weight may also affect self‐reporting in certain populations, leading to regional variations in prevalence. One study found that the prevalence of emotional eating especially of sweets was higher among people in rural compared to urban areas of Europe, potentially associated with worse health outcomes such as cardiometabolic disorders and especially mental health issues such as depression and suicide (Ljubičić et al., 2023). On the other hand, emotional eating may be prevalent in HICs due to the higher exposure to dietary triggers through food marketing and easy access to convenience, calorie‐dense foods (Finlay et al., 2022).

On the other hand, the high heterogeneity could have been explained by differences in geographical regions but was not detected in our subgroup analysis due to the disproportionally larger number of studies conducted in the European Region (*n* = 9) with only one study each from the Western Pacific Region and South‐East Asian Region included.

### No moderation effects of weight status

Our findings revealed that the proportion of overweight and obesity is not a significant moderator of emotional eating prevalence. This is contradictory to previous research that showed that emotional eating is more prevalent in those with overweight and obesity (Gibson, 2012). A possible reason could be that Gibson (2012) studied a larger range of Body Mass Index (BMI) and a wide range of both healthy populations and obese populations, while our study focuses on overweight and obese populations potentially creating a ceiling effect as to how much the emotional eating prevalence can further differ in populations that already have a high BMI.

### Limitations

There were several limitations to this study. First, half of the studies were representative of the prevalence of emotional eating in the European region, limiting the generalizability of the study findings. Secondly, the included studies had flaws in reporting their methodological quality especially in terms of reporting their sampling strategies, data analysis and response rates, limiting the accuracy of our study findings. Lastly, the use of various self‐reported measures of emotional eating could have limited the accuracy of our prevalence estimation as compared to slightly more objective measures such as having a clinical evaluation, which on the other hand, poses logistical and feasibility challenges.

### Clinical significance

Understanding the prevalence of emotional eating has significant clinical implications for preventing and managing various health conditions (Brytek‐Matera, 2021; Kornacka et al., 2021). Emotional eating is strongly associated with obesity, metabolic disorders and mental health issues such as anxiety, depression and eating disorders (Dakanalis et al., 2023). For example, one study found that college students with high stress tended to have higher frequency of eating and were more likely to eat unhealthy food such as fast food, ready‐prepared meals and sugary snacks (Choi, 2020). By knowing its prevalence, clinicians and public health professionals can identify at‐risk populations and develop targeted interventions. For example, communities experiencing high levels of stress, trauma, or economic insecurity may benefit from tailored programmes that address emotional triggers and promote healthier coping mechanisms.

Prevalence data also inform preventive strategies such as encouraging emotional regulation, mindfulness, and alternative ways to manage stress, thereby reducing the risk of chronic conditions like obesity and cardiovascular diseases (Chew et al., 2022). Furthermore, understanding the extent of emotional eating helps integrate behavioural health into nutrition counselling (Serin & Şanlıer, 2018). This interdisciplinary approach allows clinicians to address the psychological and emotional drivers of eating, improving the efficacy of weight management and dietary interventions.

Regional and cultural variations in prevalence highlight the importance of culturally tailored interventions. Differences in emotional triggers, food availability and social norms surrounding eating require programmes that are sensitive to the unique needs of diverse populations (Reichenberger et al., 2020). Prevalence data also support policymakers in resource allocation, ensuring that funding is directed towards community mental health services, nutrition education programmes, or school‐based initiatives designed to foster healthy eating habits from an early age.

Finally, understanding the scale of emotional eating facilitates early detection of disordered eating behaviours, enabling timely interventions that prevent progression to more severe conditions like binge eating disorder. It also guides research and innovation, highlighting the need for effective tools to monitor and manage emotional eating. This knowledge can spur the development of digital health solutions, such as mobile apps or wearables, to provide real‐time support for individuals struggling with emotional eating.

## AUTHOR CONTRIBUTIONS

**Han Shi Jocelyn Chew:** Conceptualization; methodology; software; validation; formal analysis; data curation; investigation; resources; visualization; project administration; writing – original draft; writing – review and editing; supervision. **Rou Yi Soong:** Investigation; validation; software; formal analysis. **Wei How Darryl Ang:** Writing – review and editing; validation. **Jia Wen Ngooi:** Validation; writing – review and editing. **Jiyoung Park:** Validation; writing – review and editing. **Jenna Qing Yun Ow Yong:** Validation; software; formal analysis; investigation. **Yong Shian Shawn Goh:** Conceptualization; software; validation; formal analysis; investigation; writing – original draft; writing – review and editing; supervision.

## FUNDING INFORMATION

This research did not receive any specific grant from funding agencies in the public, commercial, or not‐for‐profit sectors.

## CONFLICT OF INTEREST STATEMENT

None.

## CONSENT FOR PUBLICATION

I hereby provide consent for the publication of the manuscript detailed above.

## Supporting information


Data S1.

## Data Availability

Available on request from the corresponding author.
