# Supplementary material for: The global prevalence of emotional eating in overweight and obese populations: A systematic review and meta‐analysis
Source: Br J Psychol. 2025 Jan 15;116(2):484–98. doi: 10.1111/bjop.12768 (PMC11984340; doi:10.1111/bjop.12768)

Table S1: Preferred Reporting Items for Systematic reviews and Meta‐Analyses (PRISMA 2020) checklist

| **Section and Topic** | **Item #** | **Checklist item** | **Location where item is reported** |
| --- | --- | --- | --- |
| **TITLE** | | |  |
| Title | 1 | Identify the report as a systematic review. | Title Page, Page 1 |
| **ABSTRACT** | | |  |
| Abstract | 2 | See the PRISMA 2020 for Abstracts checklist. | Page 1 |
| **INTRODUCTION** | | |  |
| Rationale | 3 | Describe the rationale for the review in the context of existing knowledge. | Page 2-3 |
| Objectives | 4 | Provide an explicit statement of the objective(s) or question(s) the review addresses. | Page 3 |
| **METHODS** | | |  |
| Eligibility criteria | 5 | Specify the inclusion and exclusion criteria for the review and how studies were grouped for the syntheses. | Page 3-4 |
| Information sources | 6 | Specify all databases, registers, websites, organisations, reference lists and other sources searched or consulted to identify studies. Specify the date when each source was last searched or consulted. | Page 6  Page 6 |
| Search strategy | 7 | Present the full search strategies for all databases, registers and websites, including any filters and limits used. | Table S2 |
| Selection process | 8 | Specify the methods used to decide whether a study met the inclusion criteria of the review, including how many reviewers screened each record and each report retrieved, whether they worked independently, and if applicable, details of automation tools used in the process. | Page 3 |
| Data collection process | 9 | Specify the methods used to collect data from reports, including how many reviewers collected data from each report, whether they worked independently, any processes for obtaining or confirming data from study investigators, and if applicable, details of automation tools used in the process. | Page 3, 6 |
| Data items | 10a | List and define all outcomes for which data were sought. Specify whether all results that were compatible with each outcome domain in each study were sought (e.g. for all measures, time points, analyses), and if not, the methods used to decide which results to collect. | Page 6 |
|  | 10b | List and define all other variables for which data were sought (e.g. participant and intervention characteristics, funding sources). Describe any assumptions made about any missing or unclear information. | Page 6 |
| Study risk of bias assessment | 11 | Specify the methods used to assess risk of bias in the included studies, including details of the tool(s) used, how many reviewers assessed each study and whether they worked independently, and if applicable, details of automation tools used in the process. | Page 6-7 |
| Effect measures | 12 | Specify for each outcome the effect measure(s) (e.g. risk ratio, mean difference) used in the synthesis or presentation of results. | Page 7 |
| Synthesis methods | 13a | Describe the processes used to decide which studies were eligible for each synthesis (e.g. tabulating the study intervention characteristics and comparing against the planned groups for each synthesis (item #5)). | Page 7 |
|  | 13b | Describe any methods required to prepare the data for presentation or synthesis, such as handling of missing summary statistics, or data conversions. | Page 7 |
|  | 13c | Describe any methods used to tabulate or visually display results of individual studies and syntheses. | Page 7 |
|  | 13d | Describe any methods used to synthesize results and provide a rationale for the choice(s). If meta-analysis was performed, describe the model(s), method(s) to identify the presence and extent of statistical heterogeneity, and software package(s) used. | Page 7 |
|  | 13e | Describe any methods used to explore possible causes of heterogeneity among study results (e.g. subgroup analysis, meta-regression). | Page 7 |
|  | 13f | Describe any sensitivity analyses conducted to assess robustness of the synthesized results. | Page 7 |
| Reporting bias assessment | 14 | Describe any methods used to assess risk of bias due to missing results in a synthesis (arising from reporting biases). | Page 7 |
| Certainty assessment | 15 | Describe any methods used to assess certainty (or confidence) in the body of evidence for an outcome. | Page 7 |
| **RESULTS** | | |  |
| Study selection | 16a | Describe the results of the search and selection process, from the number of records identified in the search to the number of studies included in the review, ideally using a flow diagram. | Page 5, 8 |
|  | 16b | Cite studies that might appear to meet the inclusion criteria, but which were excluded, and explain why they were excluded. | Page 5, 8  Table S3 |
| Study characteristics | 17 | Cite each included study and present its characteristics. | Table 1 |
| Risk of bias in studies | 18 | Present assessments of risk of bias for each included study. | Table S4 |
| Results of individual studies | 19 | For all outcomes, present, for each study: (a) summary statistics for each group (where appropriate) and (b) an effect estimate and its precision (e.g. confidence/credible interval), ideally using structured tables or plots. | Page 8-10 |
| Results of syntheses | 20a | For each synthesis, briefly summarise the characteristics and risk of bias among contributing studies. | Page 8-9 |
|  | 20b | Present results of all statistical syntheses conducted. If meta-analysis was done, present for each the summary estimate and its precision (e.g. confidence/credible interval) and measures of statistical heterogeneity. If comparing groups, describe the direction of the effect. | Page 9-11 |
|  | 20c | Present results of all investigations of possible causes of heterogeneity among study results. | Page 8-9 |
|  | 20d | Present results of all sensitivity analyses conducted to assess the robustness of the synthesized results. | Page 9 |
| Reporting biases | 21 | Present assessments of risk of bias due to missing results (arising from reporting biases) for each synthesis assessed. | Page 9 |
| Certainty of evidence | 22 | Present assessments of certainty (or confidence) in the body of evidence for each outcome assessed. | Page 8-9 |
| **DISCUSSION** | | |  |
| Discussion | 23a | Provide a general interpretation of the results in the context of other evidence. | Page 11-13 |
|  | 23b | Discuss any limitations of the evidence included in the review. | Page 13-14 |
|  | 23c | Discuss any limitations of the review processes used. | Page 13-14 |
|  | 23d | Discuss implications of the results for practice, policy, and future research. | Page 12-14 |
| **OTHER INFORMATION** | | |  |
| Registration and protocol | 24a | Provide registration information for the review, including register name and registration number, or state that the review was not registered. | Title Page |
|  | 24b | Indicate where the review protocol can be accessed, or state that a protocol was not prepared. | Title Page |
|  | 24c | Describe and explain any amendments to information provided at registration or in the protocol. | NA |
| Support | 25 | Describe sources of financial or non-financial support for the review, and the role of the funders or sponsors in the review. | Title Page |
| Competing interests | 26 | Declare any competing interests of review authors. | Title Page |
| Availability of data, code and other materials | 27 | Report which of the following are publicly available and where they can be found: template data collection forms; data extracted from included studies; data used for all analyses; analytic code; any other materials used in the review. | Title Page |

Table S2: Search Strategy

| **Database** | **Index and keyword terms** | **Results (first search)** | **Results (updated search)** |
| --- | --- | --- | --- |
| *PubMed* | (((emotional OR stress OR boredom) AND (eating OR eater)) AND (overweight[Title/Abstract] OR obesity[Title/Abstract] OR obese[Title/Abstract] OR weight gain[Title/Abstract] OR weight loss[Title/Abstract] OR weight maintenance[Title/Abstract] OR body mass index[Title/Abstract])) AND (Prevalence[Title/Abstract] OR incidence[Title/Abstract] OR proportion[Title/Abstract] OR epidemiology[Title/Abstract]) | 695 | 105 |
| *EMBASE* | (emotional OR stress OR boredom) AND (eating OR eater) AND (overweight:ab,ti OR obesity:ab,ti OR obese:ab,ti OR 'weight gain':ab,ti OR 'weight loss':ab,ti OR 'weight maintenance':ab,ti OR 'body mass index':ab,ti) AND (prevalence:ab,ti OR incidence:ab,ti OR proportion:ab,ti OR epidemiology:ab,ti) | 838 | 30 |
| *The Cochrane Library* | emotional OR stress OR boredom in All Text AND eating OR eater in All Text AND overweight OR obesity OR obese OR weight gain OR weight loss OR weight maintenance OR body mass index in Title Abstract Keyword AND Prevalence OR incidence OR proportion OR epidemiology in Title Abstract Keyword - (Word variations have been searched) | 224 | 37 |
| *CINAHL* | AB ( emotional OR stress OR boredom ) AND AB ( eating OR eater ) AND AB ( overweight OR obesity OR obese OR weight gain OR weight loss OR weight maintenance OR body mass index ) AND AB ( Prevalence OR incidence OR proportion OR epidemiology ) | 197 | 15 |
| *PsycINFO* | ((emotional or stress or boredom) and (eating or eater)).af. and (overweight or obesity or obese or weight gain or weight loss or weight maintenance or body mass index).ab. and (Prevalence or incidence or proportion or epidemiology).ab. | 241 | 72 |
| *Scopus* | ( TITLE-ABS-KEY ( emotional OR stress OR boredom ) AND TITLE-ABS-KEY ( eating OR eater ) AND TITLE-ABS-KEY ( overweight OR obesity OR obese OR weight AND gain OR weight AND loss OR weight AND maintenance OR body AND mass AND index ) AND TITLE-ABS-KEY ( prevalence OR incidence OR proportion OR epidemiology ) ) | 230 | 35 |
| *Web of Science* | emotional OR stress OR boredom (Topic) and eating OR eater (Topic) and overweight OR obesity OR obese OR weight gain OR weight loss OR weight maintenance OR body mass index (Abstract) and Prevalence OR incidence OR proportion OR epidemiology (Abstract) | 580 | 83 |
|  | | **3,005** | **392** |
| **Total** | | **3397** | |

Table S3: List of excluded articles and reasons

| **No.** | **Author, Year** | **Title of Article** | **Reason for exclusion** |
| --- | --- | --- | --- |
| 1 | Ali et al., 2023 | The Impact of Negative Emotions on Binge Eating and BMI Among Medical Students | No prevalence data on emotional eating |
| 2 | Belyh et al., 2023 | Features of eating behavior in adolescents | Non-English language (Russian) |
| 3 | Benbaibeche et al., 2023 | Emotional and external eating styles associated with obesity | No prevalence data on emotional eating |
| 4 | Bober et al., 2023 | Eating Behaviours of Polish and Portuguese Adults-Cross-Sectional Surveys | No prevalence data on emotional eating |
| 5 | Chen et al., 2023 | Differential pathways to disordered eating for immigrant and native adolescents in Taiwan | No prevalence data on emotional eating |
| 6 | Constant et al., 2024 | Exploring sub-threshold food addiction in adult patients with severe obesity: A cross-sectional analysis | No prevalence data on emotional eating |
| 7 | Dakanalis et al., 2023 | The Association of Emotional Eating with Overweight/Obesity, Depression, Anxiety/Stress, and Dietary Patterns: A Review of the Current Clinical Evidence | Secondary research |
| 8 | Díaz et al., 2023 | Macronutrient Intake, Sleep Quality, Anxiety, Adherence to a Mediterranean Diet and Emotional Eating among Female Health Science Undergraduate Students | No prevalence data on emotional eating |
| 9 | Hu et al., 2023 | Associations of COVID-19 Lockdowns on Eating Behaviors and Body Mass Index in Patients with a History of Bariatric Surgery: a Cross-Sectional Analysis | No prevalence data on emotional eating |
| 10 | Ilić et al., 2023 | Motivations Associated with Food Choices among Adults from Urban Setting | No prevalence data on emotional eating |
| 11 | Isakova et al., 2023 | Assessment of Eating Behavior in Patients with Metabolic Disorders | Non-English language (Russian) |
| 12 | Ishak et al., 2023 | BMI-for-age z-score and psychological distress associated with disordered eating: A comparative study among Indonesian and Malaysian urban female adolescents | No prevalence data on emotional eating |
| 13 | Kim et al., 2023 | A validation study of the Dutch Eating Behavior Questionnaire for Children (DEBQ-C) for children and adolescents with obesity in South Korea | Conference abstract |
| 14 | Lopez-Cepero et al., 2023 | Higher Engagement in Emotional and Uncontrolled Eating is Associated with Higher Prevalence of Obesity and Type 2 Diabetes among Adults in Puerto Rico | Conference abstract |
| 15 | Makki et al., 2024 | Depressive, Anxiety, and Stress Symptoms Among Schoolgirls With Disordered Eating Behaviors | No prevalence data on emotional eating |
| 16 | McAulay et al., 2023 | Eating disorder features in bipolar disorder: clinical implications | No prevalence data on emotional eating |
| 17 | Meseri et al., 2023 | A factor that can yield to eating attitude disorders in university students: Self-esteem | No prevalence data on emotional eating |
| 18 | Miggantz et al., 2023 | Characteristics of Active Duty Service Members Referred to the Navy's Weight-Management Program | No prevalence data on emotional eating |
| 19 | Panariello et al., 2023 | Eating Disorders and Disturbed Eating Behaviors Underlying Body Weight Differences in Patients Affected by Endometriosis: Preliminary Results from an Italian Cross-Sectional Study | No prevalence data on emotional eating |
| 20 | Rosenqvist et al., 2023 | Stress-induced eating and drinking and their associations with weight among women and men during 30-year follow-up | No prevalence data on emotional eating |
| 21 | Sosa-Cordobés et al., 2024 | Emotional eating in university students: a cross-sectional and comparative study in the Iberian Peninsula | Non-English language (Spanish) |
| 22 | Tang et al., 2023 | Characteristics of Malaysian 16-year-old girls who reported regular binge eating episodes and the associated risk factors: a descriptive study | No prevalence data on emotional eating |
| 23 | Warschburger et al., 2023 | Stability and longitudinal association between Body Mass Index and maladaptive eating behaviors in older adults: Results from the NutriAct Family Study (NFS) | No prevalence data on emotional eating |
| 24 | Wu et al., 2023 | Excessive gestational weight gain and emotional eating are positively associated with postpartum depressive symptoms among taiwanese women | No prevalence data on emotional eating |

Table S4: JBI Critical Appraisal Checklist for Studies Reporting Prevalence Data

| **Criteria** | Tuncer et al., 2020 | Constanta et al., 2018 | Chacko et al., 2015 | Grajek et al., 2022 | Schnettler et al., 2021 | Barak et al., 2021 | Sze et al., 2021 | Wong et al., 2020 | Al-Musharaf, 2020 | Mohapatra et al., 2021 | Skolmowska et al., 2022 | Jääskeläinen et al., 2014 | Aagaard et al., 2023 | Bizjak et al., 2023 | Gazdzinska, 2024 | Constant, 2023 | Hawash, 2024 | Zuhair, 2024 |
| --- | --- | --- | --- | --- | --- | --- | --- | --- | --- | --- | --- | --- | --- | --- | --- | --- | --- | --- |
| **1** | + | + | + | + | + | + | + | + | + | + | + | + | + | + | + | - | + | + |
| **2** | + | - | + | +/- | + | +/- | - | - | - | - | + | +/- | - | - | - | - | + | + |
| **3** | + | +/- | +/- | + | + | + | + | + | + | + | + | + | - | + | + | + | + | + |
| **4** | + | + | + | + | + | + | + | + | + | + | + | + | + | + | + | + | + | + |
| **5** | - | + | + | + | +/- | - | + | +/- | - | - | + | + | +/- | - | + | + | +/- | + |
| **6** | + | + | + | + | +/- | + | + | + | + | + | + | + | + | + | + | + | + | + |
| **7** | + | + | + | + | + | + | - | - | + | + | + | + | +/- | + | +/- | + | + | +/- |
| **8** | + | + | + | + | + | + | + | + | + | + | + | + | + | + | + | + | + | + |
| **9** | +/- | + | - | + | - | + | +/- | + | - | +/- | + | - | + | - | + | + | - | + |
| **Overall** | 7 / 9 = 78% | 7 / 9 = 78% | 7 / 9 = 78% | 8 / 9 = 89% | 6 / 9 = 67% | 7 / 9 = 78% | 6 / 9 = 67% | 6 / 9 = 67% | 6 / 9 = 67% | 6 / 9 = 67% | 9 / 9 = 100% | 7 / 9 = 78% | 5 / 9 = 56% | 6 / 9 = 67% | 7 / 9 = 78% | 7 / 9 = 78% | 7 / 9 = 78% | 8 / 9 = 89% |

1 = Was the sample frame appropriate to address the target population?; 2 = Were study participants sampled in an appropriate way?; 3 = Was the sample size adequate?; 4 = Were the study subjects and the setting described in detail?; 5 = Was the data analysis conducted with sufficient coverage of the identified sample?; 6 = Were valid methods used for the identification of the condition?; 7 = Was the condition measured in a standard, reliable way for all participants?; 8 = Was there appropriate statistical analysis?; 9 = Was the response rate adequate, and if not, was the low response rate managed appropriately?; + = Yes; +/- = Unclear; - = No; N.A. = Not Applicable

Figure S5: Funnel Plot


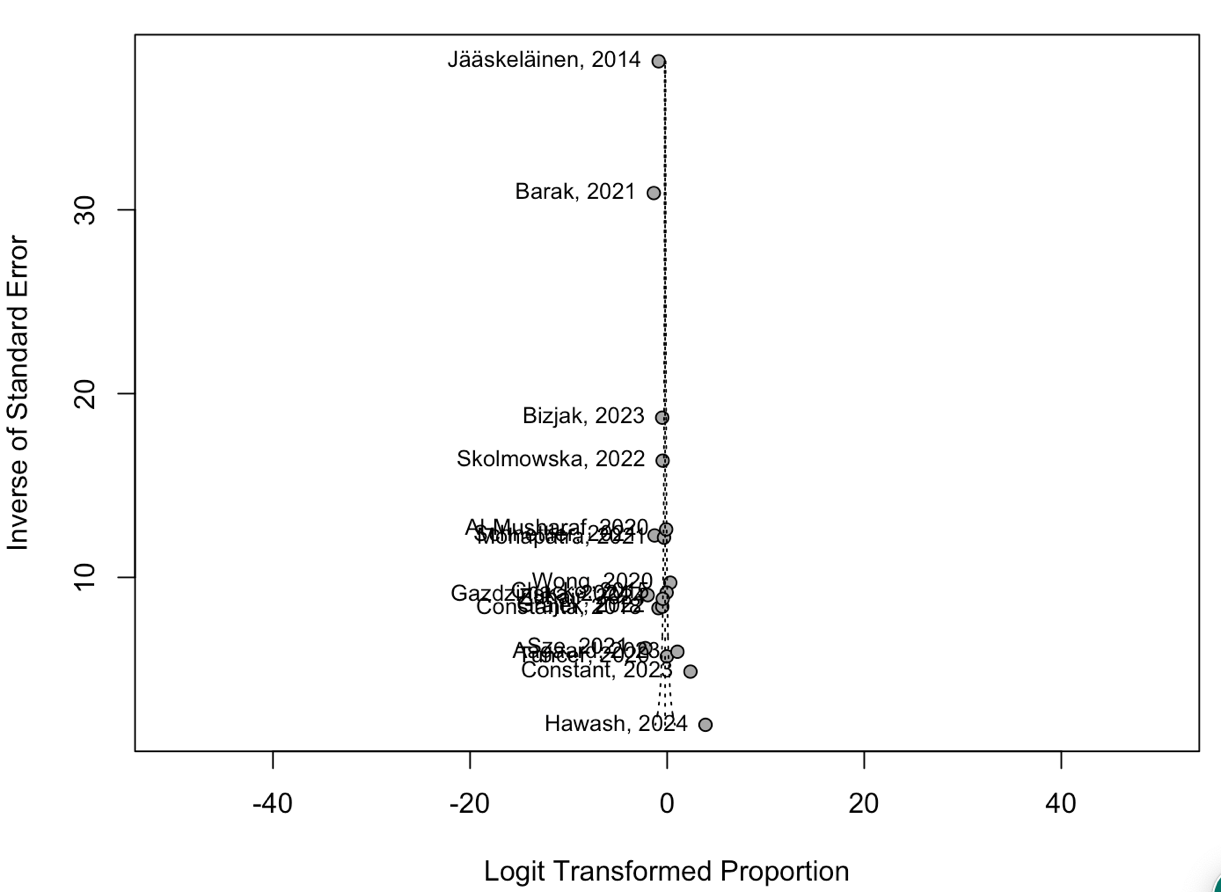

Supplement: Supplementary file 1 — Data S1. [file BJOP-116-484-s001.docx]
